# Pediatric chronic pain programs: current and ideal practice

**DOI:** 10.1097/PR9.0000000000000613

**Published:** 2017-08-21

**Authors:** Jordi Miró, Patrick J. McGrath, G. Allen Finley, Gary A. Walco

**Affiliations:** a Unit for the Study and Treatment of Pain—ALGOS, Chair in Pediatric Pain URV-Fundación Grünenthal; b Research Center for Behavior Assessment (CRAMC), Department of Psychology, Institut d'Investigació Sanitària Pere Virgili, Universitat Rovira i Virgili, Tarragona, Catalonia, Spain; c Centre for Pediatric Pain Research, IWK Health Centre and Dalhousie University, Halifax, NS, Canada; d Department of Anesthesiology & Pain Medicine, Seattle Children's Hospital, University of Washington School of Medicine, Seattle, WA, USA

**Keywords:** Pediatric chronic pain, Treatment, Chronic pain programs, Survey

## Abstract

**Introduction:**

The treatment of youth with chronic pain has improved in recent years. However, because pediatric chronic pain programs are not governed by international standards, the development and implementation of new initiatives may be limited.

**Objectives:**

The objectives of this study were to identify the features of programs as they exist at present and to determine what features they should have in an ideal state.

**Methods:**

A web-based international survey was used to collect information. The survey contained 86 questions seeking respondent professional demographic data and information about the pain program with which the respondent was affiliated at the time (program organization, types of pain problem treated, professionals involved, services provided, size of the program, research, professional training, public education and advocacy, and funding sources).

**Results:**

Respondents were 136 pediatric pain experts representing different specialties located in 12 countries. Most respondents indicated that ideal programs would have a multidisciplinary staff; provide a wide range of treatments for different chronic pain problems; integrate research, formal clinical training of specialists, and public education and advocacy into their activities; and be an accredited part of the public health system.

**Conclusions:**

The results of this survey may be useful for health care professionals interested in treating chronic pain in children and adolescents and for policy makers concerned with improving the care given to these children and their families.

## Abstract 2

This report may be useful for health care professionals and policy makers concerned with improving the care of children with chronic pain and their families.

## 1. Introduction

Chronic pain in children and adolescents is a significant problem worldwide, with prevalence rates varying (from 6% to 37%) as a function of the reporting period used and how chronic pain is defined.^16^ The treatment of pediatric pain has improved significantly in recent years,^6,27,28^ largely because of the recognition that pain is a complex experience resulting from the interaction of biological, emotional, cognitive, behavioral, and social factors.^18^ Although further progress is always possible,^25^ the improvements in the prevention and control of acute and procedural pain, for example, have been significant.^8,24,26^ Even though there has also been clear progress in addressing chronic pain,^21,22^ the breakthroughs in research are not always reflected in daily clinical activity.^4,17^ However, there is consensus that the treatment of pediatric chronic pain requires an interdisciplinary approach,^1,10,20^ and a number of programs worldwide have reported successful results applying such models.^5,8,11,15,29^ Such programs are relatively sparse, however, and information about how models have been and may be generalized ideally on an international level is lacking.

Some descriptions of ideal pediatric pain programs have been published. For example, Berde et al.^3^ provided some general recommendations for the design of a pediatric pain center (for acute and chronic pain) on the basis of the program at Boston Children's Hospital. Peng et al.^23^ went into greater detail about the activities involved in pediatric chronic pain programs, describing the services offered by 5 Canadian multidisciplinary pain treatment facilities. Although these and other similar publications^2,9,29^ are useful, they are limited in scope, lack details, and are not specific enough to provide guidance on how to organize a program for treating pediatric chronic pain.

While these program descriptions have elements in common, there is no consensus to guide standards on the structure and functioning of these programs. Without perspective on key elements of what to do, how to do it, and by whom, it is difficult to develop or improve programs, including the integration of research and clinical practice. The objectives of this study were to (1) identify features of current pediatric chronic pain programs and (2) describe such features in an ideal situation.

## 2. Methods

### 2.1. Procedure

The survey was conducted in the last 3 months of 2015. At that time, there were no international lists of multidisciplinary pain programs available, only some regionally such as in the USA (http://americanpainsociety.org/uploads/get-involved/PediatricPainClinicList_Update_2.10.15.pdf), Canada (http://www.canadianpaincoalition.ca/downloads/pain_clinics.pdf), and Australia (www.apsoc.org.au/facility-directory). Consequently, we contacted potential respondents by (1) posting a note in the newsletter of the International Association for the Study of Pain (IASP) Special Interest Group on Pain in Childhood; (2) sending a series of e-mails to subscribers of 2 pediatric pain Listservs subscribed internationally—one sponsored by the IASP (iasp-Pain in childhood) and the other hosted by Dalhousie University, the PEDIATRIC-PAIN email discussion list (http://pediatric-pain.ca/pediatric-pain-mailing-list/); and (3) sending messages to selected colleagues asking them to forward the request to others working in the field. The note and messages mentioned the objectives of the web survey, explicitly stating that they were “to identify the characteristics of these (pediatric chronic pain) programs as they exist, but also to learn about ideal characteristics.”

The message that potential participants received included a link to the questionnaire. Approval from the IWK Health Centre Research Ethics Board was requested and obtained by way of a waiver, as this project was regarded as program evaluation and not human research. The participants first answered questions about the pediatric pain program to which they were affiliated at the time of the survey, and then they were asked about their ideal pediatric pain program.

### 2.2. Measurement

The survey questionnaire contained 86 questions in English on a wide range of issues related to the areas of roles and processes. The questions were specifically developed for this survey by the authors. The first set of questions focused on the respondents' professional demographic information, and then covered the following areas of the pain program to which they were affiliated at the time: organization of the program (eg, types of pain problem treated, professionals involved, and size of the program), research, training or education of professionals, public education and advocacy, types of treatment provided, delivery of services, and funding sources (the questionnaire is available from the authors on request). The questions were designed to identify the basic characteristics of the programs and the kind of issues that were thought to be of interest for potential users of this information. Once the questions had been generated, the survey was piloted with 5 clinicians and researchers in chronic pediatric pain to identify potential difficulties.

### 2.3. Data analysis

The information in the questionnaires was coded and scored by a research assistant, and the results are presented as median and percentage scores.

## 3. Results

### 3.1. Characteristics of participants

**Figure 1.**
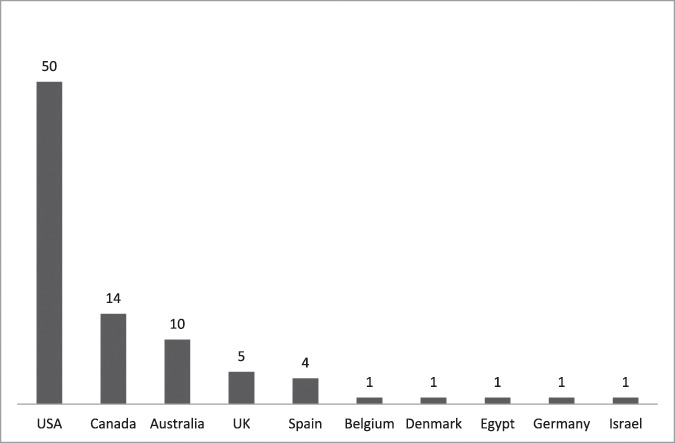
Country of program being reported. Number of respondents = 88 (65%).

A total of 136 professionals, mostly psychologists, anesthesiologists, and nurses, representing 12 countries answered the survey (Table 1). Most were from the English-speaking world: USA, Canada, Australia, and UK. Respondents were mostly female, with specialized training in pediatric pain at the doctoral level. The participants reported being active in publishing in this area; responses identifying the quantity of articles published in the 5-year period ranged from 2 to 40 (0–10 articles: N = 11; 10–20: N = 39; 20–30: N = 26; >30: N = 21) (Fig. 1).

**Table 1:**
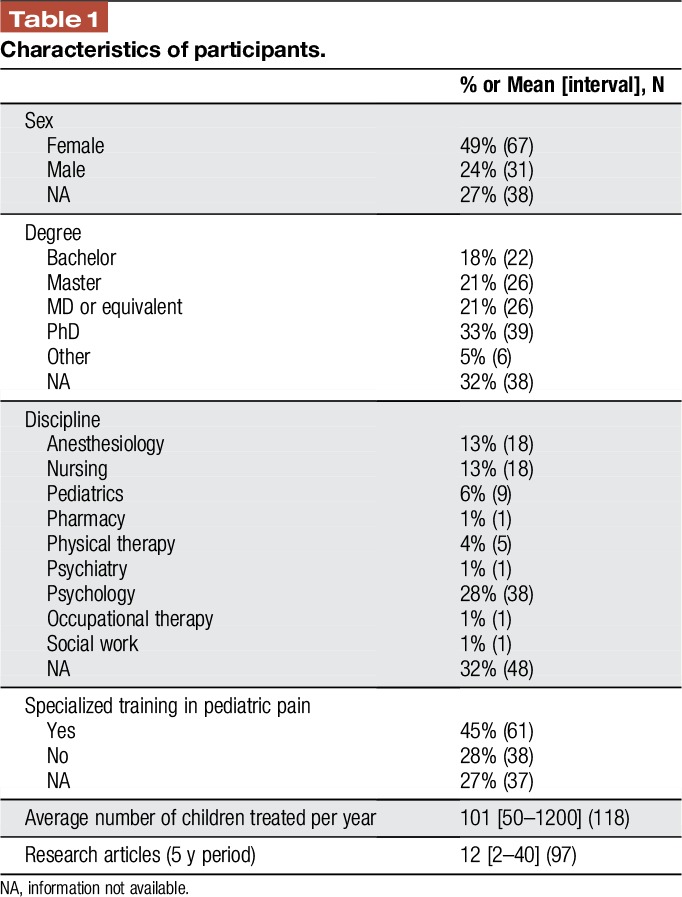
Characteristics of participants.

### 3.2. Main features of existing pediatric chronic pain programs

**Figure 4.**
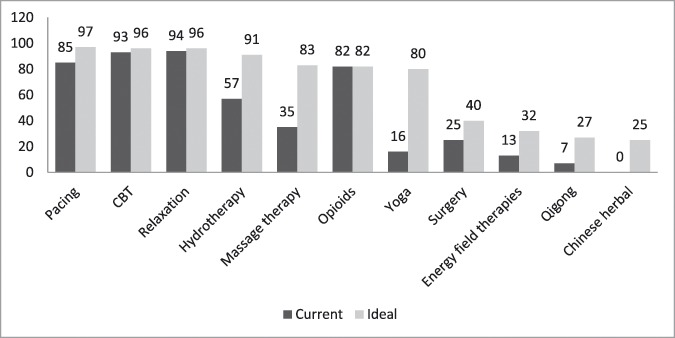
Types of treatment. Data are in percentages; Number of respondents = 79 (58%). CBT, cognitive behavioral therapy.

Most programs were specialized units. Many were led by an anesthesiologist, although they were multidisciplinary in nature, with anesthesiologists, nurses, physiotherapists, and psychologists being the professionals most frequently involved. Academic activities, including research and training, were integral to many of the programs. A little over half of the respondents reported that public education and advocacy was a feature of their program. Most of the programs functioned as outpatient services. The modal psychological treatment being offered was cognitive behavioral therapy (Fig. 4). Most of these were public—government funded—programs, with a mean of almost 5 full-time employees (Table 2).

**Table 2:**
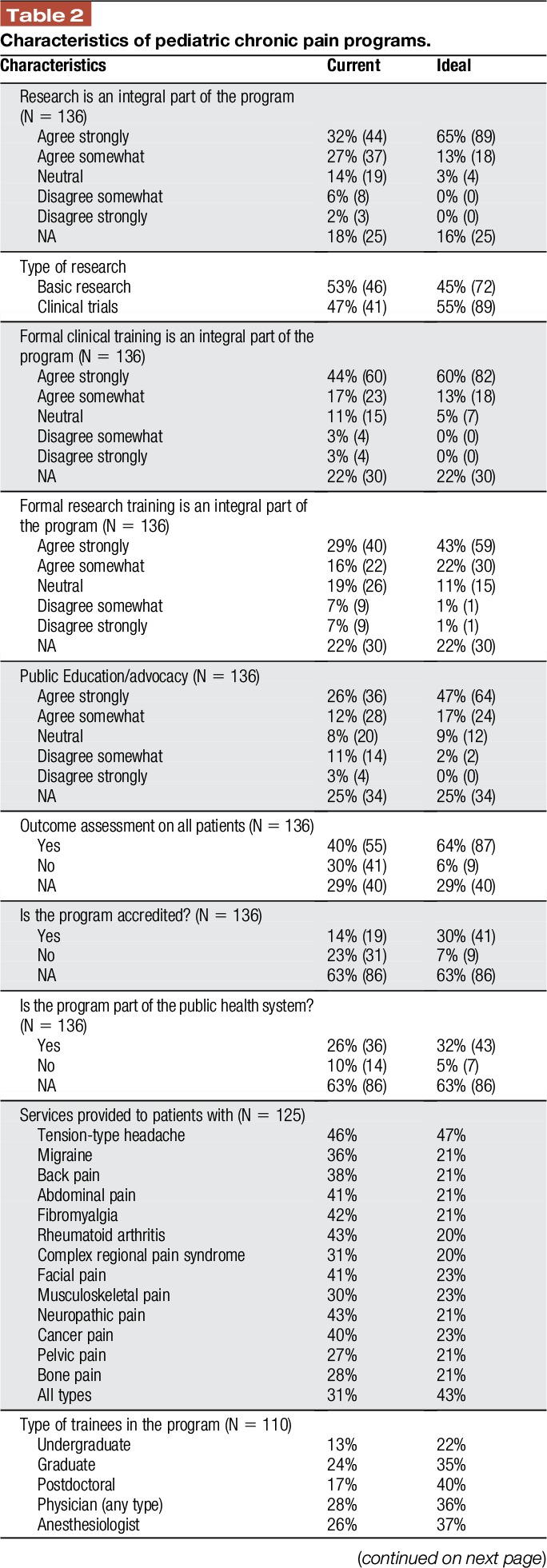

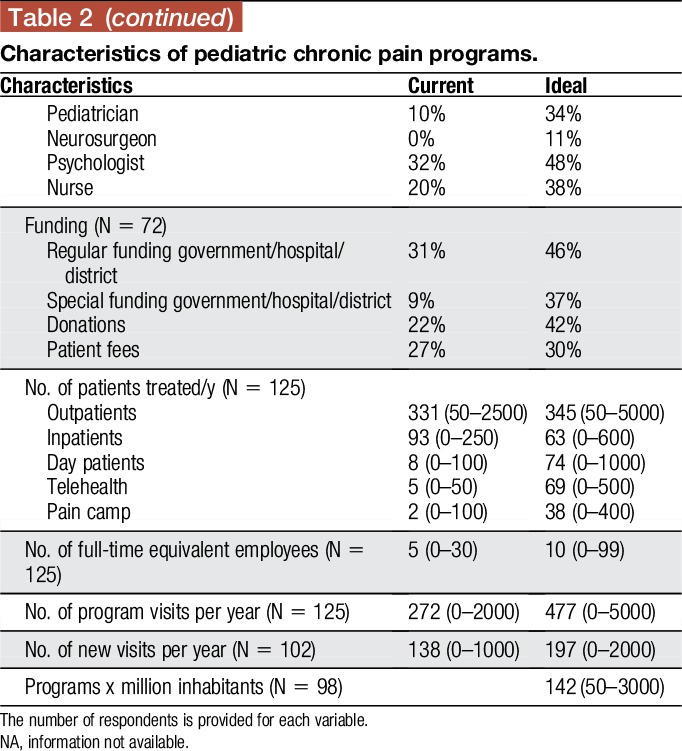
Characteristics of pediatric chronic pain programs.

### 3.3. Ideal features of pediatric chronic pain programs

For the most part, respondents felt that the program leader could be from any discipline (34%), but they thought that personal characteristics and the willingness to be the leader were more important than the specific background or training received.

The ideal program should give an important role to both research and clinical training (a total of 80% and 73% of respondents, respectively, agreed with these statements). Also important, but less so, was public education and advocacy (Table 2). Most respondents also believed that programs should be an accredited part of a public health system. Participants in the survey also considered that programs should have more full-time employees (78% responded that more staff would be needed). Although pediatric chronic pain programs differ in size and scope, and thus the number of professionals required might also differ, it was thought that this increase would allow them to help many more children. Almost half the respondents (43%) reported that programs should deal with all chronic pain problems and not focus on a single specific type of pain (an additional 27% selected 5 pain problems or more).

**Figure 2.**
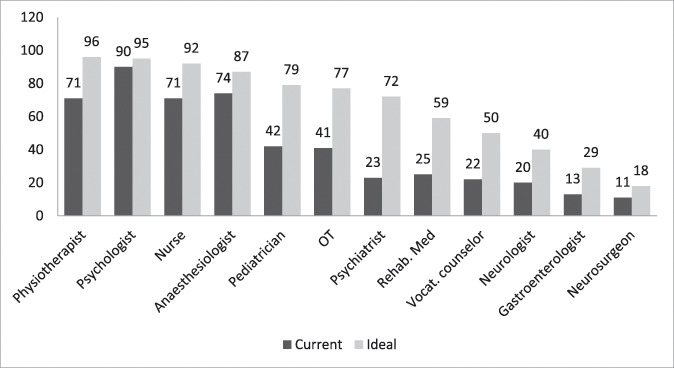
Program staffing. Data are in percentages; Number of respondents = 98 (72%). OT, occupational therapist.

Figure 2 summarizes the types of professional that the respondents considered to be important for a program. Interestingly, anesthesiologists are in fourth position, although they were the second choice as the best leaders. However, more than a third of the participants in the survey felt that leaders could come from any discipline (Table 2).

**Figure 3.**
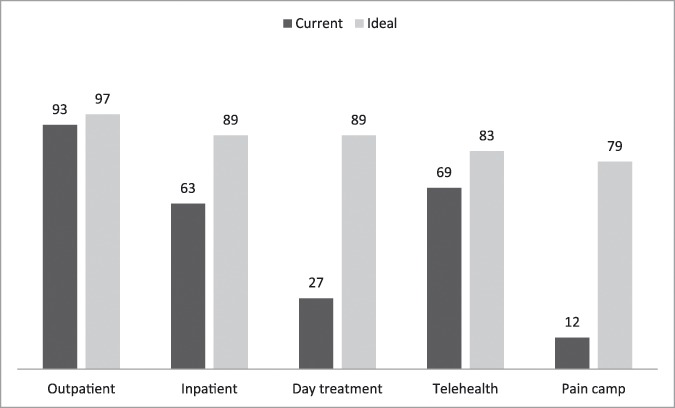
Delivery of services. Data are in percentages. Number of respondents = 102 (75%).

Ideally, programs would deliver their treatments at least on an outpatient basis, although respondents also considered inpatient services, daily treatment, and services provided through information and communication technologies to be important. Intensive educational and treatment programs, typically a day treatment format, were seldom provided but appealed to a large percentage of respondents (Fig. 3). According to respondents, these programs should provide a wide range of treatments (Fig. 4). In this ideal situation, all respondents agreed that patients should be assessed by the team (multidisciplinary approach), not just by one professional. The question about the accreditation of the program received fewer responses (41%) than the other questions in the survey, making the information difficult to interpret.

## 4. Discussion

The objectives of this study were to identify current features of programs treating children and adolescents with chronic pain, as well as to solicit international expert opinion on ideal features of these programs.

The results of this survey show that most current chronic pain treatment programs function as outpatient services, are multidisciplinary in nature, are based on a biopsychosocial model of pain, and provide cognitive behavioral therapy–based psychological treatments. These programs highlight research with training and education of future professionals and advocacy being integral parts.

Major differences were found between current and ideal scenarios in several areas. The first of these was the leader's specialty. In an ideal scenario, most participants felt that the program leaders could be from any discipline. Personal characteristics and the willingness to be the leader were deemed more important than specific background or training. The second disparity focused on types of treatments provided. In an ideal situation, programs would offer a wide range of treatments, far broader than what is currently provided. Third, participants asserted that programs ought to be accredited more than they are at present (31% vs 14%, respectively), carry out more research (96% vs 73%), provide formal clinical training for pain specialists (94% vs 78%), be part of a public health care system (43% vs 36%), and increase staff size (10 vs 5 full-time equivalent employees).

This comparison of the current and ideal scenarios might lead to the erroneous conclusion that the situation is close to ideal in many areas. However, there are still countries where pediatric chronic pain treatment is not recognized, and there are no specific programs or protocols for pediatric chronic pain problems. For example, Spain has no multidisciplinary programs to attend to the needs of young people with chronic pain,^19^ although the prevalence of chronic pain among the general population has been reported as reaching 37% of children and adolescents between 8 and 16 year old.^12^ Although this may be an extreme example, it is not the only country under these circumstances and it certainly reflects the lack of pain programs for those who need such specialized help.

Although interdisciplinary pediatric chronic pain programs are generally regarded as the best form of treatment,^1^ there is very little information, including empirical data, available on the key features of pediatric chronic pain programs. In fact, there is no information on the availability of treatment programs for young people with chronic pain around the world. In 2009, an international task force from the IASP developed recommendations for wait times^14^ and suggested that acute painful conditions should be treated immediately, severe conditions with risk of chronicity should be treated within 1 week, progressive pain with a duration of 6 months or less ought to be treated within 1 month, and cases of persistent long-term pain without significant progression should receive treatment within 8 weeks of referral. Given that very few pain programs are currently available, it is highly unlikely that these recommendations are met. The more waiting times exceed the recommendations, the more the quality of life of these children (and of their families) deteriorate. Furthermore, the problems become more difficult to treat while quality of life continues to decline.

The results of this study also show that not all programs are staffed with the professionals required for multidisciplinary pain centers, according to IASP recommendations,^13^ which include physicians, nurses, physical therapists, and psychologists. Thus, although current pediatric chronic pain programs provide a range of focal treatments, they need a wider variety of professionals, if they are to provide the best treatment possible. The participants in this survey considered that, in an ideal situation, these programs should offer a wide range of treatments. However, this is the best situation possible. The suggestions made by this international and multidisciplinary group of experts would have to be implemented in specific contexts, and therefore adapted to the needs and resources available. For example, future studies should examine what combination of treatments and services are considered to be ideal for various specific pain diagnoses. Likewise, future studies could also explore the minimum services that should be provided by these programs. It could be hypothesized that the higher percentages of responses in this study reflect services that are considered to be the highest priority. However, this is no more than a reasonable hypothesis, and this issue needs to be investigated further.

This study had a number of limitations that may be borne in mind when interpreting the results. Although we did everything we could to reach as many professionals involved in pediatric chronic pain programs as possible, the sample of participants might be biased in ways of which we are unaware. For example, we received a good number of responses from professionals working in the USA, which may reflect a language bias or the fact that such programs are more prevalent in that country. This may have been addressed if we had provided the questionnaire in languages other than English. Although English is a common language of interaction and communication among professionals and we received responses from non–English-speaking countries, professionals who are not proficient in English may have self-excluded.

We tried to recruit only pediatric pain specialists as respondents. We used strategies to invite such experts to participate, and the information provided about years of expertise and publications on pediatric pain generally make us confident in the validity of their responses. However, we had no way to assure that all were pediatric pain specialists. Besides, the respondent may or may not have accurately described the status quo of the care for the majority of patients. Moreover, we know nothing about the nonrespondents and have no way to ascertain if there was a significant cadre of pain professionals who we simply did not reach.

Although we did all that we could to eliminate responses which duplicate the information on the programs reported, some respondents did not provide the name of the program, so some sites may be overrepresented. In addition, a high percentage of respondents did not respond to all the questions in the survey. Thus, we used data provided and treated blank fields as missing data. Because this study was merely descriptive, no additional methods were used to address missing data.

Despite these limitations, the results of this survey may help guide the field further by providing support and rationale for the form programs should take. This survey provides valuable information for health care professionals, particularly for those who are interested in treating pediatric chronic pain, as it provides important information about the current and ideal features of pediatric chronic pain programs. However, we trust that the results may also be useful to policymakers, administrators, and others responsible for making decisions about promoting pediatric chronic pain programs in places where they are lacking and underscore the need for improved access to treatment programs around the globe.

The lack of progress and knowledge translation in the management of chronic pain, particularly in the development of programs for young people, may be due to various factors. The highly complex nature of the experience, as in the case of pediatric chronic pain, is probably at the heart of the problem. Nevertheless, the lack of guides for creating and implementing programs based on the knowledge available might also be at least partially responsible.

No single formula can be applied to all hospitals, clinics, or countries, as health care systems vary from country to country, but the information contained in this article may be useful for those planning to develop a multidisciplinary pain program for pediatric chronic pain or even for those that are already established and are seeking avenues for change and improvement.

A significant percentage of respondents (30%) agreed that, in an ideal situation, pediatric chronic pain programs ought to be accredited. However, standards are as yet unavailable, and there is no international accrediting body. Thus, one important avenue for knowledge translation and improvement of pediatric chronic pain management would be to develop a set of agreed standards that programs must comply with to be accredited by a national or international body.

Promoting change, and particularly significant and long-lasting change, involves going beyond simply giving guidelines or training initiatives. Organizational and intellectual changes are also needed (how things are conceptualized, the attitudes of the personnel involved, etc.), and that is no easy task. The information contained in this study, and in others to be produced in the future, could help those who have started to see the need for change to envisage the possibility of developing and implementing a pediatric chronic pain program in their hospitals.

## Disclosures

The authors have no conflict of interest to declare.

This work has been partly supported by Obra Social de La Caixa; the Foundation “La Marató de TV3,” the Spanish Ministry of Economy and Competitiveness (PSI2015-70966-P and PSI2016-82004-REDT), and the European Regional Development Fund (ERDF). J. Miró's work is supported by the Institució Catalana de Recerca i Estudis Avançats (ICREA-Acadèmia) and Fundación Grünenthal.
